# Pennsylvania

**Published:** 1899-04

**Authors:** 


Secretary.

### PENNSYLVANIA STATE VETERINARY MEDICAL ASSOCIATION.

PRESIDENT JOBSON called the seventeenth annual meeting to order March 7th, at 10.30 A.M., at the Veterinary Department of the University of Pennsylvania. On roll-call by Dr. Jacob Helmer, Recording Secretary, the following members answered to their names at the several sessions of the Convention: Drs. J W. Adams, Philadelphia, Pa.; F. S. Allen, Philadelphia, Pa.; Francis Bridge, Philadelphia, Pa.; Henry Bowers, Philadelphia, Pa.; J. F. Butterfield, Montrose, Pa.; U. S. G. Bieber, Kutztown, Pa.; W. G. Benner, Doylestown, Pa.; C. W. Boyd, Allegheny, Pa.; Raymond Barber, Newtown, Pa.; M. E. Conard, West Grove, Pa.; A. O. Cawley, Milton, Pa.; Ardemus Diemer, Spring City, Pa.; Warren T. Edwards, Downingtown, Pa.; Robert Formad, Philadelphia, Pa.; J. C. Foelker,

Allentown, Pa.; J. T. Ferley, Jr., Philadelphia, Pa.; Howard B. Felton, Philadelphia, Pa.; W. Horace Hoskins, Philadelphia, Pa.; Jacob Helmer, Scranton, Pa.; S. J. J. Harger, Philadelphia, Pa.; J. D. Houldsworth, Manayunk, Pa.; H. D. Hackler, Philadelphia, Pa.; C. W. Holly, Muncy, Pa.; George B. Jobson, Franklin, Pa.; W. S. Kooker, Philadelphia, Pa.; L. O. Lusson, Ardmore, Pa.; J. Stewart Lacock, Allegheny, Pa.; J. C. Michener, Colmar, Pa.; C. J. Marshall, Philadelphia, Pa.; James R. Mahaffy, Wilmington, Del.; Otto G. Noack, Reading, Pa.; James T. McAnulty, Philadelphia, Pa.; J. H. Oyler, Harrisburg, Pa.; Leonard Pearson, Philadelphia, Pa.; E. M. Ranck, Philadelphia, Pa.; James B. Rayner, West Chester, Pa.; A. W. Radley, Bethlehem, Pa.; N. E. Reinhart, Pottstown, Pa.; John B. Raynor, Branchtown, Pa.; N. Rectenwald, Pittsburg, Pa.; W. H. Ridge, Trevose, Pa.; W. L. Rhoads, Lansdowne, Pa.; B. F. Senseman, Philadelphia, Pa.; G. K. Swank, Mauch Chunk, Pa.; E. E. Terry, Holmesburg, Pa.; H. W. Turner, Lahaska, Pa.; B. M. Underhill, Media, Pa.; Charles T. Williams, Philadelphia, Pa.

Among the visiting members of the veterinary and medical profession were to be noted: Drs. J. N. Becker, Palmyra, Pa.; H. S. Butterfield, Montrose, Pa.; H. S. Drake, Leesburg, Va.; Mr. F. Enge, Philadelphia, Pa.; Drs. W. H. Fry, Pine Grove Mills, Pa.; P. B. Fernsler, Lebanon, Pa.; L. B. Horner, Woodstown, N. J.; L. P. Hurley, Hopewell, N. J.; Mr. John Norman Henry, Philadelphia, Pa.; Drs. W. B. Montgomery, Chestnut Hill, Philadelphia, Pa.; L. E. Mead, Tunkhannock, Pa.; James M. Mecray, Maple Shade, N. J.; A. R. May, Boiling Springs, Pa.; W. Walter Martin, Philadelphia, Pa.; J. Cheston Morris, Philadelphia, Pa.; Henry D. Martein, Philadelphia, Pa.; John H. McNeall, Philadelphia, Pa.; T. B. Rogers, Woodbury, N. J.; J. J. Rectenwald, Mt. Oliver, Pa.; F. H. Schnieder, Ashland, Pa.; A. T. Sellers, Camden, N. J.; E. K. Shaub, Lancaster, Pa.; W. C. Smith, Philadelphia, Pa.; Mr. J. J. Teufel, Philadelphia, Pa.; Drs. J. W. Van Horn, Montoursville, Pa.; Charles R. Walter, Conyngham, Pa.

A large body of the veterinary students were present at the several sessions: Harry E. Bender, Harry E. Bassler, E. L. Cornman, C. L. Colton, F. S. Carlisle, R. Y. Davison, Charles S. Gelbert, Jr., Samuel H. Gilland, G. W. Horner, Herbert Hoopes, J. W. Hayman, William Hughes, M. Jacob, A. G. Kern, C. F. Keiter, John P. Miller, O. M. Norton, E. W. Newcomer, Lawrence A. Nolan, W. E. Phillips, Reuben Rivers, C. S. Shore, Edward Seidel, Oscar Seeley, B. H. Tallman, N. S. Townsend, Frederick Taylor, F. J. Woodward, Harry W. Watson, Thomas White, F. H. Willett.

The reading of the minutes of the Pittsburg meeting in September and their approval followed.

The election of officers for the ensuing year resulted in the selection of the following members of the Association for the respective places: President, Dr. J. Curtis Michener, Colmar; First, or Eastern Vice-President, Dr. S. J. J. Harger, Philadelphia; Second, or Central Vice-President, Dr. Otto G. Noack, Reading; Third, or Western Vice-President, Dr. J. C. McNeil, Pittsburg; Recording Secretary, Dr. C. J. Marshall, Philadelphia; Corresponding Secretary, Dr. W. L. Rhoads, Lansdowne; Treasurer, Dr. Francis Bridge, Philadelphia. Board of Trustees: George B. Jobson, W. Horace Hoskins, James W. Sallade, W. H. Ridge, and James B. Rayner.

The following applications for membership were received: Drs. E. K.

Shaub, Lancaster, graduate of American Veterinary College; C. W. Holly, Muncy, graduate of Ontario; C. M. Strickler, Greencastle, graduate of Ontario; U. S. G. Bieber, Kutztown, graduate of American Veterinary College; John R. Courtright, Clark's Green, National Veterinary College; James R. Mehaffey, Wilmington, Del., Veterinary Department of the University of Pennsylvania—all of whom were favorably reported and elected except one name, which was laid over for further examination.

One violation of the Code of Ethics was reported and referred to the Board of Trustees, with power to act on its removal before reporting to the Association for final action. In the absence of Members McNeil and Sallade, of the Board of Trustees, the President appointed Drs. Lusson and Allen to act with the other members of the Board.

One member from Delaware was elected, having a license of the State Board of Pennsylvania and practising in the southern part of the State.

The Treasurer's report exhibited a balance of $239.02, and the sum of $532 due from members.

Letters and telegrams of regret at their inability to be present, assigning sickness, death in family, etc., as potent reasons, were read from Members Irons, Heckenberger, Lushington, Miller, Thomas B. Rayner, M. J. Collins, M. W. Heck, D. C. Stanton, Harry D. Hanson.

The report of Chairman, Dr. H. B. Felton, of the Committee on Intelligence and Education, was one of the best ever presented to the Association; and though the author had just emerged from a long and trying period of illness, he exhibited the most gratifying evidence of keeping in close touch at all times with the profession and its work, and won a very hearty vote of thanks for his much-appreciated efforts. His report was supplemented by additional ones from Member Kooker, of the committee.

An early adjournment the first day enabled the members and visitors to participate in a clinical demonstration of a number of operations by Prof. S. J. J. Harger, of surgical interference for cribbing. Methods of casting and securing animals for operations, for relief of stringhalt, caponizing, etc., by Drs. Adams, Rectenwald, and others.

Luncheon was served each day at the University restaurant, and supper for all visitors and others, so as to keep the members together. The Philadelphia veterinarians, under the direction and management of Dr. C. J. Marshall, tendered this courtesy to the members of the convention.

An evening session was held the first day, at which the following subjects were considered: "The Value of a Wholesome Meat Supply," by Dr. Alfred Stengel, of the William Pepper Clinical Laboratory; "Diseases of Animals Transmissible to Man," illustrated by lantern-slides, by Dr. M. P. Ravenel, of the State Livestock Sanitary Board; "Municipal Slaughter-houses, and their Need," by Dr. Leonard Pearson, State Veterinarian.

More than one hundred were present at the evening session, and for two hours the speakers received the utmost attention to their unusually entertaining and instructive addresses.

*Wednesday*, *March* 8, 1899.—President Jobson called the meeting to order at 10.20 A.M. There was a very much increased attendance.

Dr. Robert Formad was called upon for his "Demonstration of Tumors from a Pathological Stand-point." Surrounded by preserved specimens of every kind, shape, and form of tumors gathered from the various museums of the University of Pennsylvania, the subject was entered upon with all the enthusiasm and interest that only those can display who are thoroughly schooled in their subjects; and for forty-five minutes the members were graphically told of the clinical aspects of these pathological changes, and assurdely every one present realized for once that even so abstruse a subject could be presented in an attractively instructive manner, and warmly showed their appreciation of the demonstrator's successful work.

Dr. W. Horace Hoskins reported at some length as chairman of the Committee on Legislation, reviewing the several acts of interest to veterinarians now pending before the State Legislature.

Dr. Leonard Pearson, chairman of the Committee on Sanitary Science and Police, made an extemporaneous report of much interest, reviewing briefly the records of work in this direction by the Livestock Sanitary Board, the recent contributions to the clinical treatment of milk-fever and contagious abortion, commenting upon what had been accomplished in dealing with tuberculosis and the value of the present restrictive measures. Dr. C. J. Marshall, of the same committee, made an excellent statistical report relative to the prevalence, etc., of osteoporosis, with a summary of the views of a large number of the profession throughout the State as to the chief points of its development, nature, etc.

So many matters of importance were presented by the various committee reports and in the papers and County Secretaries' reports that the President, under a resolution, appointed a committee of five to draft suitable resolutions expressive of the Association's interest and views, and named Drs. W. Horace Hoskins, W. S. Kooker, Leonard Pearson, J. Stewart Lacock, and W. H. Ridge.

Corresponding Secretary Rhoads, in his usually pertinent report, aroused the interest of the convention in his reference to the delinquent members and his appeal for increased attendance, and wound up with a gratifying presentment of the solid financial basis of the Association. The names of those reported in arrears were referred to the Board of Trustees, which body recommended their suspension from the rolls. This recommendation was qualified by the appointment of a committee to make a special appeal to these members before final action would become of force. The resignation of Dr. W. A. Heckenberger, of Catasauqua, in view of his declining years, was received with expressions of regret on all sides when the Board of Trustees recommended its acceptance; and before acting on this recommendation there was a general appeal for Member J. C. Foelker to visit him and seek his withdrawal of the resignation, all of which was generously accorded in.

Treasurer Bridge rose to present a question of importance, to his mind, in that he desired the Association to exact a bond in accordance with the By-Laws. He preferred to give one, and at his earnest request the matter was placed in the hands of the Board of Trustees, with power to act.

Then followed papers by Dr. J. Curtis Michener on "Prognosis." Always a practical writer and full of hard sense in his excellent suggestions, his paper was well received.

In the absence of Dr. Lushington, his paper on "Stock Farm Veterinary Practice as a Post-graduate Course" was read by Secretary Rhoads, and elicited much interest, as the writer has recently been called to teach veterinary science at a Virginia Agricultural and Industrial School.

Dr. J. J. Butterfield, on "Castration of Cryptorchids"—a subject which has so often been surrounded with so much delusion and deceit—was treated of in an open and definite manner.

Dr. Otto Noack, in his paper on "Azoturia," dealt with the recent studies of the nature of this malady and the clinical treatment these deductions indicated.

Dr. N. E. Reinhart, in his paper on "Tuberculosis in Dairy Cattle, and How Shall we Get Rid of It," strongly urged the value of tuberculin tests and the elimination of the diseased ones. He spoke in a sanguine manner of the prospects of its ultimate elimination from our dairies through effective inspection and the maintenance of the State law establishing a barrier to its entrance from outside sources, through animals brought in to replenish our dairies and breeding farms.

The papers of Drs. J. B. Irons, R. G. Rice, and W. S. Phillips on "Fracture of Tibia," "The Use of Chloral Hydrate in Indigestion and Colic," and "Tracheotmy in Laryngitis and Choking," respectively, were read by the Secretary.

The Committee on Resolutions presented the following:

*Whereas*, The State of Pennsylvania has, during the past three years, made an energetic effort to suppress tuberculosis of cattle, and has made much progress in this work, and it is important that the permanency of this work shall be insured, so that as our herds are freed from disease they shall be kept pure; and

*Whereas*, It has been shown that tuberculosis prevails among the dairy cattle of every State from which Pennsylvania draws her supply, and that our herds are protected to a very important degree by the law requiring an inspection of all dairy cows and cattle for breeding purposes coming into Pennsylvania from other States; and

*Whereas*, A bill has been introduced into the House of Representatives by Hon. W. H. Rosenberry[1] for the purpose of repealing the bill conferring this valuable protection; be it hereby

*Resolved*, That we protest earnestly and vigorously against the Rosenberry bill, on the ground that it is opposed to the livestock and health interests of the State; that it will expose us to the inroads of tuberculous cattle from every side, and that there is no public demand for the repeal of this bill; but, on the contrary, that the protection the existing law affords is generally appreciated and its continuation desired. Be it further

*Resolved*, That we hereby pledge ourselves to use every honorable means to defeat this dangerous measure.

*Indorsement of the Sexton Bill.*

Recognizing the grave danger of the spread of the diseases recognized and designated by the State Livestock Sanitary Board as contagious or infectious, resulting from improper disposition of the carcasses, parts thereof, or animals, or their products affected with such diseases, this committee deems it advisable for the Pennsylvania State Veterinary Medical Association to recommend to the State Senators and Representatives the passage of the bill known as the Sexton bill, which provides for the proper and sanitary disposal of the entire carcasses and products thereof of all animals known to be affected at the time of death or dying of such disease.

*Statistics of Live Stock Diseases.*

*Whereas*, A reliable collection of statistics in relation to the prevalence of the infectious diseases is much needed by the profession of this State; be it

[1] This bill, we are glad to say, has been withdrawn by the author, as the oppositon to the same from the stockowners was almost unanimous.—Ed.

*Resolved,* That the Morrison bill, which provides for the collection of these statistics, is hereby approved and indorsed.

*State Laboratory.*

*Whereas,* The adoption by the State authorities of plans for the protection of the interests of all citizens has in recent years added that of looking to all the needs and the protection of the livestock interests that are so closely allied with the whole question of pure food supply; and

*Whereas,* This work has demanded that the study of these questions and diseases of livestock, which cause annually such gigantic losses to our stock owners, add hundreds to the death-roll of our people, impose endless suffering upon a large number of our citizens, not to speak of the pecuniary burdens imposed, shall be carried out under the most advanced methods for the solution of these problems; and

*Whereas,* Our State, in its characteristic progressive spirit, has established its own laboratory for this work, which has already proved its worth in shedding light upon these obscure infectious and contagious diseases, and, in the work done for their control and eradication, saved many thousands of dollars in the cost of so doing by scientific investigation and the preparation of necessary products for use in this work; be it

*Resolved,* That we recommend to the Appropriation Committee of our State Legislature the most liberal spirit toward maintaining the laboratory of our State Livestock Sanitary Board, and that an increased appropriation may be granted that its work may be extended in other directions that are of vital health and monetary importance to our people.

*State Meat Inspection.*

*Whereas,* There has been introduced in the present session of our State Legislative body a proposed measure, known as the Adams bill, to create an inspection service throughout our State of all storage stations and warehouses for the reception of meat slaughtered outside the limits of our Commonwealth, without seeking adequate provision for a similar inspection of all like places within our State where home-dressed meats are stored; and

*Whereas,* Such inspection does not insure the consumers of meat products that safety which a State inspection service should give; be it

*Resolved,* That we recommend the amendment of this bill to cover the slaughter and exposure for sale of all meats intended for consumption by our people, and of all places where meats are slaughtered, stored, or offered for sale.

*More Stringent Milk Inspection.*

*Whereas,* The inspection of milk in this State is of such vital importance we deem it advisable to encourage any measures which tend to greater security to the consumers of milk.

*Resolved,* That this inspection shall be more in the direction of the sanitary one than to multiply police measures that are oppressive, obnoxious, and lose sight of the chief ends of all such legislation, which should always be as to its purity and freedom from the dangers of an unhealthy herd and unsanitary source of production.

*State Veterinarian.*

*Whereas,* The continued valued services of our efficient State Veterinarian Dr. Leonard Pearson by the present State administration is much appreciated by the entire veterinary profession of our State; and be it

*Resolved,* That we most cordially recommend his reappointment, that the State may be more richly benefited by his intelligent, conservative, and widespread usefulness; and be it further

*Resolved,* That we look with just pride and due appreciation to the fact that every progressive State in the line of veterinary, sanitary police measures is following in the lead of this Commonwealth, and endeavoring to care for the livestock and public health interests by the adoption of similar regulations that prevail in this State; and be it further

*Resolved*, That such unsought meed of praise is the highest commendation of the wisdom of the appointing and selecting power that secures for the welfare of our people and their interests the exceptional services that have been rendered Pennsylvania by our State Veterinarian, Dr. Leonard Pearson.

*Our State Board of Health.*

*Whereas*, Our State has never accorded to our State Board of Health that full measure of support which would conserve to the best and broadest interests of our people, and has not fully recognized the efficient, earnest, and sincere services of its efficient secretary, Dr. Benjamin Lee; and

*Whereas*, We note with pleasure a proposed measure in our State Legislature to increase the salary of the secretary to a more adequate sum; be it

*Resolved*, That we most heartily indorse this just recognition of the value of his services, and we trust that the same body will grant more liberal appropriations to this Board, that we may enjoy the benefits of the greater safeguards such recognition and assistance as would thus be assured us.

*Recommendation of Rank of Second Lieutenant.*

This committee advises that the Pennsylvania State Veterinary Medical Association show its appreciation of the recognition by the Government in the granting of the salary and allowances of second lieutenant to veterinarians who enter the United States service as such, by heartily indorsing (as a body) the passage of the bill as modified; but when the absolute necessity and the enormous value of the profession to the Government in this as well as in almost all other departments is considered, and in view of the efficient work done by the American veterinarians in thoroughly eradicating from the country such a contagious disease as pleuro-pneumonia—something never before accomplished by any other country—we feel that the profession deserves the entire recognition asked, and that we should demand the rank of second lieutenant at the earliest opportunity.

*Our Esteemed Colleague.*

*Whereas*, Our esteemed colleague, Dr. Thomas B. Rayner, a member of our organization since its inception, has met again the loss by death of a member of his own family, and also that of a brother, since our last meeting; and as a result thereof been so shattered in health himself as well as the added anxiety in the serious illness of Mrs. Rayner; be it

*Resolved*, That our Association, in convention assembled, tender to him our heartfelt wishes for a speedy restoration of his health and our sincere hopes that he may be spared for many years from any added trials to the many that have been his to bear.

*American Veterinary Medical Association.*

*Whereas*, The American Veterinary Medical Association meets at New York on the 5th, 6th, and 7th of September, and it is desirable for us to take the utmost interest in this meeting.

*Resolved*, That the President ask for ten members of our Association as volunteer delegates to attend this meeting and to join with the officers of this Association in every way in our power to make this meeting the most successful one in the East for the past ten years.

---

*Whereas*, Our Association has again enjoyed the privilege and exceptional advantages of meeting at the Veterinary Department of the University of Pennsylvania;

*Resolved*, That a vote of thanks be extended to the department for this privilege; to its dean for his thoughtful provision for our comfort, entertainment, and enjoyment of all the department's facilities.

*Whereas*, The present meeting being instructive and entertaining and also of such large attendance;

*Resolved*, That a vote of thanks be extended to our officers for their energetic work, and also to our members who so ably entertained us with such valuable papers, reports, and demonstrations.

W. Horace Hoskins,
Leonard Pearson,
W. H. Ridge,
W. S. Kooker,
J. Stewart Lacock,
Committee on Resolutions.

They were all adopted by the Association and the officers directed to transmit them to those who would place them before the respective bodies and people.

After seating the newly-elected officers the place of meeting for our next semi-annual gathering was decided in favor of Scranton, after which the meeting adjourned.

